# Predictive value of soluble urokinase-type plasminogen activator receptor for mortality in patients with suspected myocardial infarction

**DOI:** 10.1007/s00392-019-01475-1

**Published:** 2019-04-16

**Authors:** Nils A. Sörensen, Julius Nikorowitsch, Johannes T. Neumann, Nicole Rübsamen, Alina Goßling, Tau S. Hartikainen, Stefan Blankenberg, Dirk Westermann, Tanja Zeller, Mahir Karakas

**Affiliations:** 1 Department of General and Interventional Cardiology, University Heart Center Hamburg Eppendorf, Martinistr. 52, 20246 Hamburg, Germany; 2 German Center for Cardiovascular Research (DZHK), Partner Site Hamburg/Kiel/Lübeck, Hamburg, Germany

**Keywords:** ACS, Mortality, Risk prediction, Soluble urokinase-type plasminogen activator receptor (suPAR)

## Abstract

**Background:**

Early risk stratification of patients with suspected acute myocardial infarction (AMI) constitutes an unmet need in current daily clinical practice. We aimed to evaluate the predictive value of soluble urokinase-type plasminogen activator receptor (suPAR) levels for 1-year mortality in patients with suspected AMI.

**Methods and results:**

suPAR levels were determined in 1314 patients presenting to the emergency department with suspected AMI. Patients were followed up for 12 months to assess all-cause mortality. Of 1314 patients included, 308 were diagnosed with AMI. Median suPAR levels did not differ between subjects with AMI compared to non-AMI (3.5 ng/ml vs. 3.2 ng/ml, *p* = 0.066). suPAR levels reliably predicted all-cause mortality after 1 year. Hazard ratio for 1-year mortality was 12.6 (*p* < 0.001) in the quartile with the highest suPAR levels compared to the first quartile. The prognostic value for 6-month mortality was comparable to an established risk prediction model, the Global Registry of Acute Coronary Events (GRACE) score, with an AUC of 0.79 (95% CI 0.72–0.86) for the GRACE score and 0.77 (95% CI 0.69–0.84) for suPAR. Addition of suPAR improved the GRACE score, as shown by integrated discrimination improvement statistics of 0.036 (*p* = 0.03) suggesting a further discrimination of events from non-events by the addition of suPAR.

**Conclusions:**

suPAR levels reliably predicted mortality in patients with suspected AMI.

**Study registration:**

http://www.clinicaltrials.gov (NCT02355457).

**Electronic supplementary material:**

The online version of this article (10.1007/s00392-019-01475-1) contains supplementary material, which is available to authorized users.

## Introduction

Risk stratification in patients presenting with suspected acute myocardial infarction (AMI) is of major clinical relevance to identify individuals at risk of death and guide further diagnostics and or therapeutic pathways [1]. The wide range of potential differential diagnoses in these patients, including harmless conditions, but also acute life-threatening diseases such as pulmonary embolism and myocardial infarction, challenges risk prediction [2, 3].

Soluble urokinase-type plasminogen activator receptor (suPAR) is the circulating form of a three-domain membrane-bound receptor. Increased suPAR blood levels are typically observed in states of inflammation and renal impairment. In patients with renal disease, suPAR reliably predicted the worsening of kidney function [4], and even seems to be directly involved in pathogenesis of chronic kidney diseases [5]. Besides, suPAR is expressed in a variety of cells [6], which play a critical role in all stages of atherogenesis—from the initiation of fatty streaks to progression of atherosclerosis and plaque destabilization [7].

Due to multiple connections in the pathophysiology and the risk factors between cardiovascular and renal diseases [8], it seems plausible that suPAR, a marker for immune activation and inflammation, may provide prognostic information in patients with suspected AMI. However, data evaluating suPAR in this high-risk population are limited [9, 10].

Therefore, it was the aim of this study to assess the association of circulating suPAR levels with mortality in a cohort of patients presenting with suspected AMI and to compare the prognostic value to an established risk stratification tool—the Global Registry of Acute Coronary Events (GRACE) score [11, 12].

## Methods

### Study population and design

The Biomarkers in Acute Cardiac Care (BACC) Study has been published before [13]. Briefly, this study prospectively recruited 1641 patients presenting with suspected AMI to the emergency department of the University Medical Center Hamburg-Eppendorf from July 2013 to April 2016. Patients were included if they presented with symptoms suggestive of AMI, were older than 18 years, were willing to participate in the study, and were able to give written informed consent. All patients underwent a routine clinical assessment as described in the current European Society of Cardiology guidelines [14]. Blood was drawn directly at admission and after 3 h. A primary diagnosis of AMI was adjudicated according to current guidelines [14] based on a high-sensitivity troponin T assay (Elecsys; Roche Diagnostics). The final discharge diagnosis used for the index event was additionally based on all available clinical, laboratory, and imaging findings in the course of the hospital stay. Two cardiologists (N.A.S. and J.T.N.) adjudicated the diagnosis independently. If the adjudicators disagreed about the diagnosis, a third cardiologist (D.W.) refereed. Moreover, AMI, including the subtypes type I and type II, was diagnosed based on the third universal definition of myocardial infarction [15].

The study complied with the Declaration of Helsinki [16], and the ethics committee of the University Medical Center Hamburg-Eppendorf approved the study protocol. All patients provided written informed consent.

### Follow-up

Patients were followed for up to 12 months to assess all-cause mortality. Patients were contacted by telephone, medical record, mail or via their general practitioner. In those patients with no available follow-up information, mortality was assessed via the local register of death. The follow-up rate after 12 months was 99.8%.

### Laboratory methods

suPAR levels were detected using the suPARnostic standard enzyme-linked immunosorbent assay (ViroGates, Birkerød, Denmark) in frozen plasma samples. The intra-assay variation was 2.75% and the inter-assay variation 9.17%.

### The GRACE score

The GRACE score is a web-based clinical application tool for 6 months of risk prediction for death in patients with ACS during 6 months post-discharge. GRACE score was calculated for every patient using the parameters such as age, history of heart failure, history of AMI, heart rate (at presentation), systolic blood pressure (at presentation), creatinine, cardiac arrest at admission, ST-segment deviation on EKG, troponin T above the 99th percentile and if percutaneous coronary intervention was performed.

### Statistical methods

The study population was described with respect to various sociodemographic and medical characteristics. Mortality rates were estimated using the method by Schemper and Smith [17].

Subjects were grouped according to quartiles of suPAR levels for displaying survival curves. The *p* value displayed on the graphics is for the log-rank test (testing the null hypothesis of equality of survival curves versus at least two of the curves are different). Additionally, the association of logarithmized suPAR levels and suPAR quartiles with mortality was assessed by Cox proportional hazards analyses (model 1). In different models, the analyses were adjusted for age (years) and sex (model 2), and in a third model, the age- and sex-adjustment were extended to conventional cardiovascular risk factors (body mass index (BMI), diabetes, smoking status, hyperlipoproteinemia, systolic blood pressure—model 3). At last, high-sensitivity troponin I was added to the model (model 4).

The accuracy of suPAR levels to assess mortality risk was compared to the GRACE score by three measures: (1) the area under the receiver-operating characteristic curve (AUC; also known as *C*-statistic or *C*-index); (2) the integrated discrimination improvement statistics (IDI), which can be viewed as the difference of the *R*2 statistic between two models, i.e. the difference in the proportion of variance explained by the two models; and (3) the net reclassification improvement (NRI) using the categories such as survival for or death within 12 months.

The assumptions for using Cox regression were tested, and no evidence of violation was found. All computations were performed with R version 3.5.1 (http://www.r-project.org/). A *p* value of < 0.05 was considered statistically significant.

## Results

### Baseline demographics

Out of 1641 individuals presenting with suspected AMI to the emergency department, suPAR results were available in 1314 patients. 1006 were diagnosed as non-AMI and 308 as having AMI (Table 1). The mean age of non-AMI patients was 63 years, while it was 68 years in AMI patients. The majority of patients was male (63.5% in non-AMI and 67.2% in AMI patients). As expected, troponin levels were higher in patients with AMI. Furthermore, patients presenting with AMI more often reported arterial hypertension and hyperlipoproteinemia. A history of coronary artery disease (CAD) was present in 39% of AMI patients; among non-AMI patients still 32% reported a history of CAD. AMI patients showed slightly higher suPAR levels compared to non-AMI patients. The difference did not reach significance (3.5 ng/ml vs. 3.2 ng/ml, *p* = 0.066) (Table 1). Characteristics of patients with type 1 and type 2 AMI are given in Table S1. Detailed characteristics and final diagnoses of patients without AMI are given in the online supplement: Among patients with non-cardiac chest pain, those with a final diagnosis of acute infection and asthma/chronic obstructive lung disease had the highest median suPAR levels (Table S2). In patients with cardiac non-coronary chest pain, highest suPAR levels were observed in heart failure patients (Table S3). Patients with unstable angina pectoris and those with stable angina pectoris had similar suPAR levels as the whole study population (Tables S4 and S5).

**Table 1:** Baseline characteristics

	All (*N* = 1314)	Non-AMI (*N* = 1006)	AMI (*N* = 308)	*p* value
Age (years)	64.0 (51.0, 75.0)	63.0 (49.0, 74.0)	68.0 (58.0, 76.0)	< 0.001
Male (%)	846 (64.4)	639 (63.5)	207 (67.2)	0.26
BMI (kg/m^2^)	26.1 (23.6, 29.3)	26.0 (23.6, 29.3)	26.2 (23.6, 29.4)	0.76
Hypertension (%)	885 (67.6)	649 (64.8)	236 (76.9)	< 0.001
Hyperlipoproteinemia (%)	502 (38.2)	359 (35.7)	143 (46.4)	< 0.001
Diabetes (%)	179 (13.8)	124 (12.5)	55 (18.1)	0.018
Former smoker (%)	386 (29.5)	297 (29.6)	89 (29.0)	0.89
Current smoker (%)	331 (25.3)	241 (24.0)	90 (29.3)	0.073
History of CAD (%)	442 (33.6)	322 (32.0)	120 (39.0)	0.028
eGFR (ml/min for 1.73 m^2^)	76.7 (59.1, 92.5)	79.6 (61.3, 94.0)	67.8 (52.2, 83.6)	< 0.001
Symptom onset ≥ 6 h (%)	693 (57.1)	535 (58.2)	158 (53.7)	0.2
hs-TnI I 0 h (ng/L)	6.8 (3.1, 21.9)	5.0 (2.5, 10.3)	88.1 (18.2, 945.7)	< 0.001
suPAR 0 h (ng/ml)	3.3 (2.3, 4.8)	3.2 (2.3, 4.7)	3.5 (2.4, 5.1)	0.066

For continuous variables, median (25th percentile, 75th percentile) is given. For binary ones, absolute and relative frequencies are shown

*BMI* body mass index, *CAD* coronary artery disease, *eGFR* estimated glomerular filtration rate, *hs-TnI* high-sensitivity troponin I, *suPAR* soluble urokinase plasminogen activator receptor

### Prediction of all-cause mortality according to suPAR levels

During a follow-up of 1 year, 68 deaths (mortality rate: 5.2%) from any cause were documented, 39 (3.9%) among non-AMI patients and 29 (9.5%) among AMI patients.

**Fig. 1:**
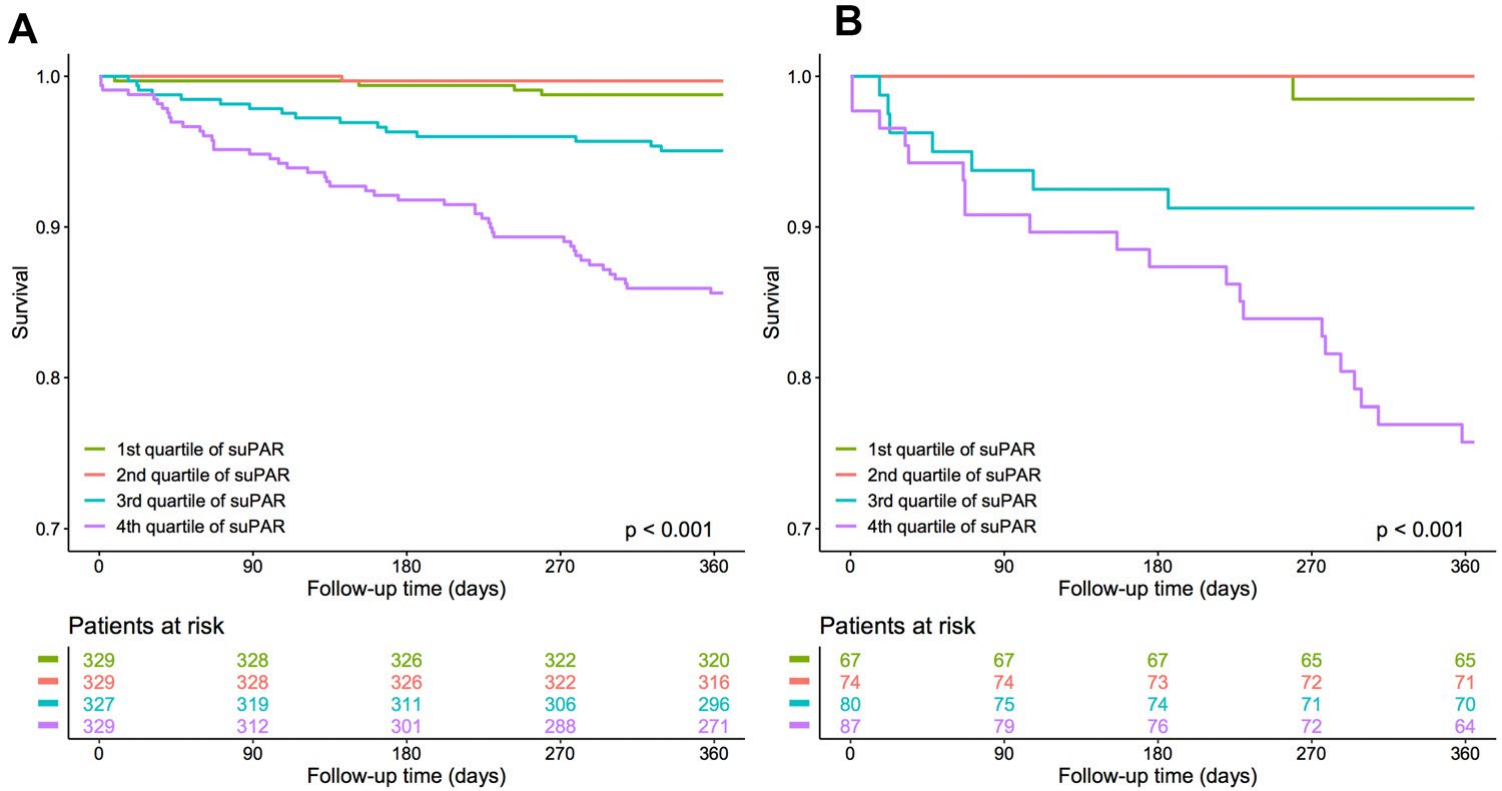
Kaplan–Meier survival analysis. Kaplan–Meier survival curves for all patients (**a**) and patients diagnosed as having AMI (**b**) stratified by quartiles of suPAR levels. *AMI* acute myocardial infarction, *suPAR* soluble urokinase plasminogen activator receptor

After categorization of all patients according to quartiles of suPAR levels, those within the fourth quartile of patients had the highest 1-year mortality (Fig. 1a). This pattern was equal when women and men were analyzed separately (Figure S1). In patients diagnosed with AMI, a similar distribution was observed (Fig. 1b). In Cox regression analyses, the unadjusted hazard ratio (HR) for 1-year all-cause mortality in all patients was 12.6 [confidence interval (CI) 4.5–34.8] for a suPAR level in the fourth quartile compared to the first quartile (*p* < 0.001). After adjustment for age, gender and common cardiovascular risk factors, the fourth quartile of suPAR still strongly predicted mortality with a HR of 4.8 (CI 1.63, 14.15; *p* = 0.0045) compared to the first quartile. After the addition of high-sensitivity troponin I to the model, suPAR still proved as an independent risk factor for overall mortality HR 4.52 (CI 1.51, 13.48; *p* = 0.007) (Table 2). Stratification by sex showed higher median suPAR levels in women compared to men (Table S6). Even after full adjustment, suPAR was associated with higher overall mortality in both sexes [HR 4.06 (1.87, 8.80), *p* < 0.001 for women and HR 3.85 (2.23, 6.66), *p* < 0.001 for men]. No interaction of suPAR-attributed risk and sex was observed (Table S7). With increasing age, median suPAR levels were higher (Table S8). Unadjusted cox regression analysis in different age groups showed weak association of suPAR levels in patients younger than 60 years [HR 1.58 (CI 0.48, 5.22), *p* = 0.46]. But in patients aged 60–70 and above 70 years suPAR was independently predictive of overall mortality, even after full adjustment: HR was 5.68 (CI 2.48, 13.01), *p* < 0.001 in patients aged 60–70 years and 4.33 (2.34, 8.00), *p* < 0.001 in patients above the age of 70 years, respectively (Table S9).

**Table 2:** Cox regression analyses of unadjusted and adjusted hazard ratios for 12-month mortality

	Model 1: HR (95% CI)	*p* value	Model 2: HR (95% CI)	*p* value	Model 3: HR (95% CI)	*p* value	Model 4: HR (95% CI)	*p* value
All patients								
Second quartile vs. first quartile of suPAR	0.25 (0.03, 2.24)	0.22	0.21 (0.02, 1.91)	0.17	0.21 (0.02, 1.85)	0.16	0.20 (0.02, 1.76)	0.15
Third quartile vs. first quartile of suPAR	4.15 (1.39, 12.41)	0.011	2.83 (0.93, 8.58)	0.067	2.75 (0.90, 8.44)	0.076	2.66 (0.86, 8.19)	0.089
Fourth quartile vs. first quartile of suPAR	12.60 (4.54, 34.97)	< 0.001	7.06 (2.46, 20.28)	< 0.001	4.80 (1.63, 14.15)	0.005	4.52 (1.51, 13.48)	0.007
Only AMI patients								
Second quartile vs. first quartile of suPAR	No events	n.a.	No events	n.a.	No events	n.a.	No events	n.a.
Third quartile vs. first quartile of suPAR	6.23 (0.77, 50.63)	0.087	4.21 (0.51, 34.71)	0.18	3.44 (0.40, 29.41)	0.26	3.37 (0.39, 29.02)	0.27
Fourth quartile vs. first quartile of suPAR	18.05 (2.43, 134.24)	0.0047	10.43 (1.37, 79.54)	0.024	6.60 (0.84, 51.72)	0.072	6.22 (0.79, 49.14)	0.083

*Model 1* unadjusted, *Model 2* adjustment for the variables age and sex, *Model 3* adjustment for the variables age, sex, diabetes, smoking, hyperlipoproteinemia and systolic blood pressure, *Model 4* adjustment for the variables age, sex, diabetes, smoking, hyperlipoproteinemia, systolic blood pressure and high-sensitivity troponin I

*HR* hazard ratio, *CI* confidence interval, *n.a.* not applicable, *suPAR* soluble urokinase plasminogen activator receptor

When only patients with AMI were considered, the fourth quartile of suPAR levels exhibited an unadjusted HR of 18 (CI 2.43, 134.24) for 1-year mortality compared to the first quartile (*p* = 0.0047). After adjustment for age and gender, a HR of 10.43 (CI 1.37, 79.54; *p* = 0.024) was observed (Table 2). Further categorization of AMI patients to type 1 and type 2 AMI showed strong association of suPAR levels and overall mortality in patients with type 1 AMI [HR after adjustment for risk factors and high-sensitivity troponin I was 3.96 (2.04, 7.71), *p* < 0.001]. For type 2 AMI patients, a suPAR-attributed risk for overall mortality was observed after adjustment for age and sex [HR 2.56 (1.02, 6.43), *p* = 0.045] but after further adjustment for cardiovascular risk factors and high-sensitivity troponin I HR did not reach significance (Table S10).

### GRACE score

**Fig. 2:**
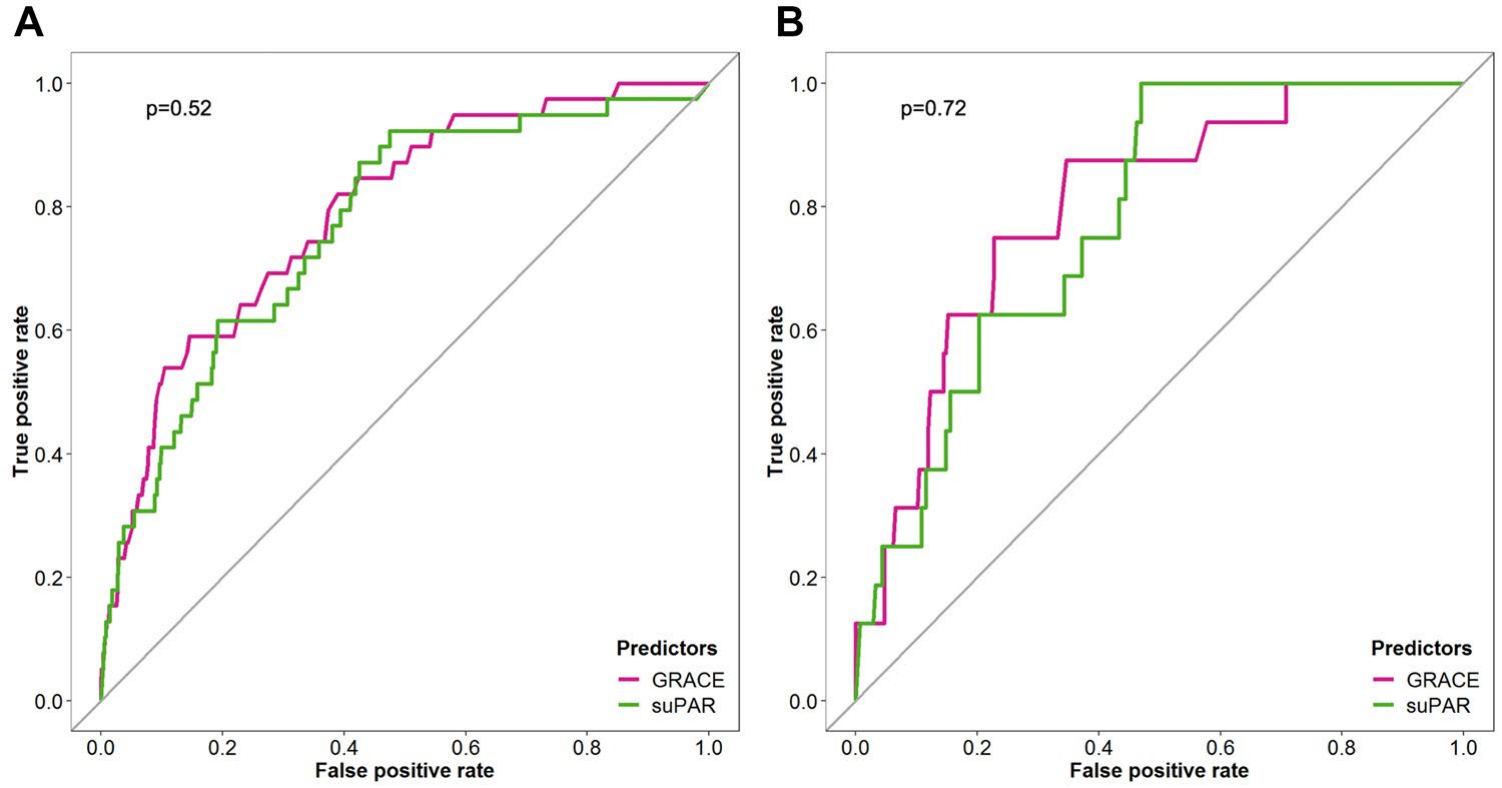
Comparison of the predictive value of suPAR and the GRACE score. ROC analyses for the prediction of 6-month mortality for GRACE score and suPAR in all patients (**a**) and only AMI patients (**b**). *GRACE* Global Registry of Acute Coronary Events, *suPAR* soluble urokinase plasminogen activator receptor

Compared to the GRACE score, the prediction of 6-month mortality in the overall cohort using suPAR as the only parameter was similar with an AUC of 0.79 (CI 0.72–0.86) and 0.77 (0.69–0.84) for the GRACE score and suPAR, respectively (*p* for difference = 0.52) (Fig. 2a). When the analyses were restricted to AMI patients, we observed an AUC of 0.80 (0.70–0.90) and 0.78 (0.69–0.87) for GRACE score and suPAR, respectively (*p* for difference = 0.72) (Fig. 2b).

The addition of suPAR information to GRACE score did not show significant NRI. While the event NRI was − 2.6% suggesting a loss of sensitivity, the non-event NRI was + 6.4% suggesting an improved specificity by the addition of suPAR compared to GRACE score alone, resulting in a positive overall NRI of 0.038 (Table 3). An improvement of the GRACE score by addition of suPAR was also confirmed by the IDI of 0.036 (*p* = 0.03), suggesting further average separation of events from non-events by addition of suPAR.

**Table 3:**
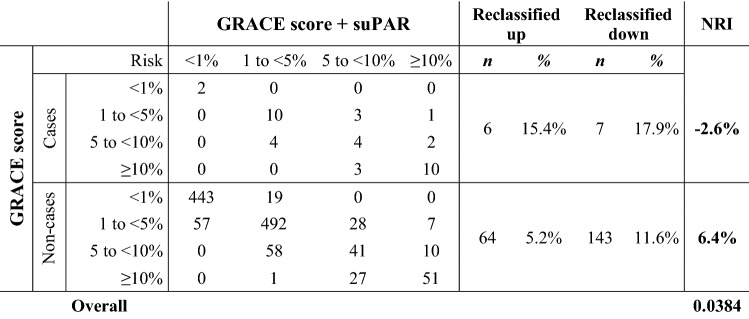
Net reclassification improvement for the combination of GRACE score and suPAR

The integrated discrimination improvement (IDI) was 0.036 (*p* = 0.03)

*GRACE* Global Registry of Acute Coronary Events, *suPAR* soluble urokinase plasminogen activator receptor, *NRI* net reclassification improvement

## Discussion

The primary finding of the present study is that the level of circulating suPAR allows for a reliable stratification for risk of 1-year mortality in a heterogenous cohort of patients presenting with suspected AMI. The accuracy of risk prediction was comparable to the established GRACE score.

Our study represents one of the largest cohorts evaluating suPAR as a prognostic marker in patients with suspected AMI so far. The observed mortality associated with suPAR levels was high: patients within the highest quartile of suPAR levels had an almost 13-fold risk of death within 12 months. Even after the adjustment for common cardiovascular risk factors, the risk was still more than 4.8-fold. After a further addition of high-sensitivity troponin I to the model, a biomarker with a strong association to fatal outcome [18], suPAR still proved as an independent predictor of overall mortality. Intriguingly, HR were significant in a subgroup analysis of women and men as well as in middle-aged and older patients, which supports the value of suPAR in an all-comer setting.

Our findings are in line with previous reports on suPAR as risk predictor: SuPAR reliably predicted mortality in the general population [19], and in patients with suspected coronary artery disease [20].

Data on the prognostic role of suPAR in cardiovascular settings are sparse. Although the prognostic efficacy of suPAR had been evaluated previously in a small Danish acute coronary syndrome (ACS) cohort, the authors were not able to compare suPAR to established risk stratification models. [21] In our study, suPAR alone achieved prognostic power comparable to the GRACE Score, a highly validated tool for predicting 6-month outcome in patients with ACS [11] and a guideline recommended score for indicating patients as low risk after MI has been ruled out [22]. Although suPAR did not improve GRACE score-based risk stratification substantially, the fact that a score, consisting of eight major risk factors including high-sensitivity troponin I, did not perform significantly better than the information derived by a single biomarker is remarkable.

Although median suPAR levels at baseline were not significantly higher in AMI patients compared to non-AMI patients and its diagnostic value is limited [23], suPAR levels were still able to predict death in patients with confirmed AMI. This was particularly true for type 1 AMI patients, which showed a strong association of suPAR with mortality. In type 2 AMI patients, a heterogenous population with a high risk of poor outcome [24, 25], suPAR-attributed risk did not reach significance after multivariate adjustment. However, the sample size of this subgroup was limited as were the reported events during 1 year of follow-up. Therefore, future studies are needed to evaluate the value of suPAR in these high-risk patients.

The concept of suPAR as a biomarker for risk stratification in suspected AMI patients is supported by an established pathophysiologic link between suPAR and atherosclerosis. The vast majority of life-limiting causes in these patients consist of atherosclerotic complications of coronary artery disease [26]. Atherosclerosis as the major underlying reason has been associated with inflammatory processes [7]. Likewise, elevated suPAR levels have typically been attributed to a state of inflammation [27] and suPAR levels measured in atherosclerotic carotid plaque were associated not only with symptoms but also with levels of proinflammatory cells, chemokines and cytokines [27, 28]. Therefore, it is presumed that proinflammatory cytokines stimulate the release of suPAR from activated neutrophils, monocytes, and endothelial cells [29]. Not surprisingly, in our analysis of patients with non-cardiac chest pain, we found the highest suPAR levels in patients with infectious disease or chronic inflammation like in asthma or chronic obstructive pulmonary disease. The fact, that we found a strong association of suPAR and mortality in AMI and non-AMI patients could imply that in both patient groups the presence of chronic inflammatory processes is linked to worse outcome. However, the underlying pathomechanisms are not well understood, which is similar for other inflammatory biomarkers, e.g. C-reactive protein or growth-differentiation factor 15—both with a strong association with cardiovascular risk and mortality [30–33].

Furthermore, suPAR meets critical requirements for a biomarker as it is relatively stable in plasma, unlike many other biomarkers it is free of circadian changes and stable during periods of acute stress [27].

### Strengths and limitations

Strengths of this study include the large cohort size of 1314 patients and a 1-year follow-up rate of more than 99.8%. However, like in most reported studies of patients with suspected AMI women were underrepresented and future studies should address sex-specific differences in suPAR related risk. Moreover, our study consisted of a large sample of AMI patients; nevertheless, a limited number of fatal events were recorded in our study. Finally, it remains unclear to what extent suPAR outperforms other inflammatory markers in predicting 1-year mortality. Specifically, high-sensitive C-reactive protein was not available in the BACC data set.

## Conclusion

Our study demonstrates that suPAR levels reliably predict mortality in the very heterogeneous group of patients presenting with symptoms suggestive of myocardial infarction in the emergency department. It, therefore, represents a valuable biomarker for risk estimation in this high-risk population.

## Electronic supplementary material

Below is the link to the electronic supplementary material.

Supplementary material 1 (DOCX 171 kb)
