# Supplementary material for: Predictive value of soluble urokinase-type plasminogen activator receptor for mortality in patients with suspected myocardial infarction
Source: Clin Res Cardiol. 2019 Apr 16;108(12):1386–93. doi: 10.1007/s00392-019-01475-1 (PMC6867986; doi:10.1007/s00392-019-01475-1)
Supplement: Supplementary file 1 — Supplementary material 1 (DOCX 171 kb) [file 392_2019_1475_MOESM1_ESM.docx]

**Online Supplement: Predictive Value of Soluble Urokinase-Type Plasminogen Activator Receptor for Mortality in Patients with suspected Myocardial Infarction**

Nils A. Sörensen, MD^1,2^, Julius Nikorowitsch, MD^1^, Johannes T. Neumann, MD^1,2^, Nicole Rübsamen, MSc^1^, Alina Goßling, MSc^1^, Tau S. Hartikainen, MD^1^, Stefan Blankenberg, MD^1,2^, Dirk Westermann, MD^1,2^, Tanja Zeller, PhD^1,2*^, Mahir Karakas, MD^1,2*^

1 Department of General and Interventional Cardiology, University Heart Center Hamburg Eppendorf, Hamburg, Germany

2 German Center for Cardiovascular Research (DZHK), Partner Site Hamburg/Kiel/Lübeck, Hamburg, Germany

*Both authors contributed equally

**Corresponding author:**

Mahir Karakas, MD

University Heart Center Hamburg, Department of General and Interventional Cardiology

Martinistr. 52, 20246 Hamburg, Germany

Phone: +49 (0) 40 7410 57975

Fax: +49 (0) 40 7410 55619

Email: m.karakas@uke.de

**Table S1: Patient characteristics of type I and type II AMI**

|  | **All types**  **(N=308)** | **Type 1**  **(N=227)** | **Type 2**  **(N=81)** | **p-value** |
| --- | --- | --- | --- | --- |
| Age (years) | 68.0 (58.0, 76.0) | 66.0 (56.0, 75.0) | 72.0 (61.0, 78.0) | 0.0062 |
| Male (%) | 207 (67.2) | 165 (72.7) | 42 (51.9) | 0.001 |
| BMI (kg/m²) | 26.2 (23.6, 29.4) | 26.3 (23.8, 29.5) | 26.2 (22.9, 28.6) | 0.26 |
| Hypertension (%) | 236 (76.9) | 171 (75.7) | 65 (80.2) | 0.49 |
| Hyperlipoproteinemia (%) | 143 (46.4) | 112 (49.3) | 31 (38.3) | 0.11 |
| Diabetes (%) | 55 (18.1) | 45 (20.2) | 10 (12.3) | 0.16 |
| Former smoker (%) | 89 (29.0) | 62 (27.4) | 27 (33.3) | 0.39 |
| Current smoker (%) | 90 (29.3) | 76 (33.6) | 14 (17.3) | 0.0085 |
| History of CAD (%) | 120 (39.0) | 95 (41.9) | 25 (30.9) | 0.11 |
| Low risk ECG (%) | 91 (30.8) | 62 (28.7) | 29 (36.7) | 0.24 |
| eGFR (mL/min for 1.73 m²) | 67.8 (52.2, 83.6) | 70.6 (52.7, 88.4) | 64.0 (51.1, 78.3) | 0.063 |
| Hs-TnI 0h (ng/L) | 88.1 (18.2, 945.7) | 136.8 (24.7, 1517.4) | 28.6 (11.9, 249.2) | <0.001 |
| suPAR 0h (ng/mL) | 3.5 (2.4, 5.1) | 3.5 (2.4, 5.1) | 3.7 (2.4, 5.2) | 0.71 |

For continuous variables median (25th percentile, 75th percentile) is given. For binary ones absolute and relative frequencies are shown. BMI: body mass index; CAD: coronary artery disease; eGFR: estimated glomerular filtration rate; hs-TnI: high-sensitivity troponin I; suPAR: soluble urokinase plasminogen activator receptor

**Table S2:** **Patient characteristics according to final diagnosis: Non-cardiac chest pain**

|  | **All non-cardiac chest pain**  **(N=500)** | **Orthopaedic (N=249)** | **Infection (N=47)** | **Epigastric (N=56)** | **Psychiatric (N=31)** | **COPD/Asthma (N=16)** | **Other (N=101)** | **p-value** |
| --- | --- | --- | --- | --- | --- | --- | --- | --- |
| Age (years) | 55.0 (44.0, 70.6) | 51.0 (40.0, 65.3) | 61.0 (48.3, 72.8) | 57.5 (47.8, 71.0) | 49.0 (45.0, 53.8) | 63.5 (51.7, 71.6) | 69.0 (52.7, 76.0) | <0.001 |
| Male (%) | 318 (63.6) | 164 (65.9) | 31 (66.0) | 33 (58.9) | 13 (41.9) | 13 (81.2) | 64 (63.4) | 0.086 |
| BMI (kg/m²) | 25.6 (22.5, 28.7) | 25.6 (22.6, 28.4) | 25.3 (21.1, 31.1) | 25.2 (22.9, 28.3) | 25.4 (21.4, 27.3) | 26.7 (23.7, 32.4) | 26.0 (23.0, 29.3) | 0.62 |
| Hypertension (%) | 246 (49.3) | 111 (44.8) | 25 (53.2) | 28 (50.0) | 9 (29.0) | 10 (62.5) | 63 (62.4) | 0.0086 |
| Hyperlipoproteinemia (%) | 118 (23.6) | 50 (20.1) | 16 (34.0) | 14 (25.0) | 9 (29.0) | 4 (25.0) | 25 (24.8) | 0.39 |
| Diabetes (%) | 39 (7.9) | 11 (4.5) | 4 (8.5) | 6 (10.7) | 4 (13.3) | 1 (6.2) | 13 (13.1) | 0.084 |
| Former smoker (%) | 124 (24.8) | 64 (25.7) | 10 (21.3) | 18 (32.7) | 5 (16.1) | 5 (31.2) | 22 (21.8) | 0.5 |
| Current smoker (%) | 152 (30.5) | 80 (32.1) | 12 (25.5) | 15 (27.3) | 11 (35.5) | 6 (37.5) | 28 (27.7) | 0.82 |
| History of CAD (%) | 86 (17.2) | 28 (11.2) | 11 (23.4) | 10 (17.9) | 4 (12.9) | 4 (25.0) | 29 (28.7) | 0.003 |
| Low risk ECG (%) | 389 (79.6) | 208 (85.2) | 31 (68.9) | 43 (79.6) | 28 (93.3) | 13 (81.2) | 66 (66.0) | <0.001 |
| eGFR (mL/min for 1.73 m²) | 86.1 (69.1, 98.9) | 90.2 (75.7, 104.3) | 85.7 (69.7, 91.2) | 85.2 (73.0, 96.0) | 87.4 (70.4, 101.4) | 91.3 (50.5, 103.7) | 72.7 (55.6, 87.4) | <0.001 |
| hs-TnI 0h (ng/L) | 3.7 (1.9, 6.6) | 3.1 (1.8, 5.9) | 5.0 (2.8, 10.4) | 3.7 (2.0, 6.3) | 2.6 (1.8, 4.8) | 4.9 (2.5, 9.2) | 5.0 (3.2, 8.9) | <0.001 |
| suPAR 0h (ng/mL) | 3.0 (2.1, 4.4) | 2.6 (1.9, 3.7) | 3.4 (2.3, 6.1) | 2.6 (2.3, 3.4) | 2.8 (2.2, 4.0) | 3.5 (2.1, 6.5) | 4.1 (2.5, 6.0) | <0.001 |

For continuous variables median (25th percentile, 75th percentile) is given. For binary ones absolute and relative frequencies are shown. BMI: body mass index; CAD: coronary artery disease; eGFR: estimated glomerular filtration rate; hs-TnI: high-sensitivity troponin I; suPAR: soluble urokinase plasminogen activator receptor

**Table S3:** **Patient characteristics according to final diagnosis: Cardiac non-coronary chest pain**

|  | **All cardiac non-coronary chest pain**  **(N=339)** | **Supraventricular tachycardia (N=87)** | **Hypertension (N=154)** | **Heart failure (N=55)** | **Other (N=43)** | **p-value** |
| --- | --- | --- | --- | --- | --- | --- |
| Age (years) | 70.0 (55.0, 77.0) | 70.0 (60.3, 76.0) | 70.5 (54.9, 77.1) | 72.0 (54.2, 77.0) | 55.0 (45.0, 76.5) | 0.025 |
| Male (%) | 199 (58.7) | 54 (62.1) | 84 (54.5) | 41 (74.5) | 20 (46.5) | 0.02 |
| BMI (kg/m²) | 26.5 (24.1, 30.4) | 26.9 (24.7, 29.8) | 26.6 (24.5, 30.4) | 25.8 (23.4, 31.1) | 25.2 (23.2, 30.5) | 0.4 |
| Hypertension (%) | 261 (77.4) | 56 (64.4) | 134 (88.2) | 46 (83.6) | 25 (58.1) | <0.001 |
| Hyperlipoproteinemia (%) | 127 (37.5) | 26 (29.9) | 62 (40.3) | 29 (52.7) | 10 (23.3) | 0.008 |
| Diabetes (%) | 57 (17.2) | 8 (9.3) | 21 (14.0) | 19 (35.2) | 9 (21.4) | <0.001 |
| Former smoker (%) | 108 (32.0) | 29 (33.3) | 51 (33.1) | 18 (33.3) | 10 (23.3) | 0.63 |
| Current smoker (%) | 54 (16.0) | 10 (11.5) | 20 (13.0) | 13 (24.1) | 11 (25.6) | 0.048 |
| History of CAD (%) | 104 (30.7) | 18 (20.7) | 45 (29.2) | 28 (50.9) | 13 (30.2) | 0.002 |
| Low risk ECG (%) | 155 (46.4) | 14 (16.3) | 105 (69.5) | 10 (18.2) | 26 (61.9) | <0.001 |
| eGFR (mL/min for 1.73 m²) | 71.3 (53.8, 87.3) | 67.3 (54.6, 84.7) | 73.3 (59.6, 86.6) | 59.2 (37.3, 79.5) | 78.2 (60.7, 101.4) | <0.001 |
| hs-TnI 0h (ng/L) | 6.9 (3.2, 15.0) | 9.0 (4.4, 19.2) | 4.8 (2.8, 8.5) | 14.3 (7.4, 47.9) | 7.3 (2.9, 24.3) | <0.001 |
| suPAR 0h (ng/mL) | 3.6 (2.7, 5.4) | 3.4 (2.6, 5.2) | 3.5 (2.6, 4.7) | 5.4 (3.4, 7.2) | 3.5 (2.3, 4.8) | <0.001 |

For continuous variables median (25th percentile, 75th percentile) is given. For binary ones absolute and relative frequencies are shown. BMI: body mass index; CAD: coronary artery disease; eGFR: estimated glomerular filtration rate; hs-TnI: high-sensitivity troponin I; suPAR: soluble urokinase plasminogen activator receptor

**Table S4:** **Patient characteristics according to final diagnosis: Unstable Angina**

|  | **Unstable**  **Angina pectoris**  **(N=156)** |
| --- | --- |
| Age (years) | 68.0 (57.0, 75.0) |
| Male (%) | 116 (74.4) |
| BMI (kg/m²) | 26.7 (24.7, 29.8) |
| Hypertension (%) | 132 (85.2) |
| Hyperlipoproteinemia (%) | 107 (68.6) |
| Diabetes (%) | 27 (17.6) |
| Former smoker (%) | 59 (38.1) |
| Current smoker (%) | 34 (21.9) |
| History of CAD (%) | 123 (78.8) |
| Low risk ECG (%) | 91 (60.3) |
| eGFR (mL/min for 1.73 m²) | 76.2 (59.0, 91.9) |
| hs-TnI 0h (ng/L) | 7.8 (3.9, 14.3) |
| suPAR 0h (ng/mL) | 3.5 (2.3, 4.8) |

For continuous variables median (25th percentile, 75th percentile) is given. For binary ones absolute and relative frequencies are shown. BMI: body mass index; CAD: coronary artery disease; eGFR: estimated glomerular filtration rate; hs-TnI: high-sensitivity troponin I; suPAR: soluble urokinase plasminogen activator receptor

**Table S5:** **Patient characteristics according to final diagnosis: Stable Angina**

|  | **Stable**  **Angina pectoris (N=11)** |
| --- | --- |
| Age (years) | 65.0 (55.0, 76.8) |
| Male (%) | 6 (54.5) |
| BMI (kg/m²) | 25.6 (23.5, 27.4) |
| Hypertension (%) | 10 (90.9) |
| Hyperlipoproteinemia (%) | 7 (63.6) |
| Diabetes (%) | 1 (9.1) |
| Former smoker (%) | 6 (54.5) |
| Current smoker (%) | 1 (9.1) |
| History of CAD (%) | 9 (81.8) |
| Low risk ECG (%) | 2 (18.2) |
| eGFR (mL/min for 1.73 m²) | 64.8 (52.8, 74.0) |
| hs-TnI 0h (ng/L) | 9.9 (5.3, 13.3) |
| suPAR 0h (ng/mL) | 3.0 (2.4, 4.9) |

For continuous variables median (25th percentile, 75th percentile) is given. For binary ones absolute and relative frequencies are shown. BMI: body mass index; CAD: coronary artery disease; eGFR: estimated glomerular filtration rate; hs-TnI: high-sensitivity troponin I; suPAR: soluble urokinase plasminogen activator receptor

**Table S6: suPAR levels in women and men**

|  | **All (N=1314)** | **Women (N=468)** | **Men (N=846)** | **p-value** |
| --- | --- | --- | --- | --- |
| Age (years) | 64.0 (51.0, 75.0) | 68.0 (53.0, 77.0) | 62.0 (50.0, 73.0) | <0.001 |
| hs-TnI 0h (ng/L) | 6.8 (3.1, 21.9) | 6.1 (2.7, 21.5) | 7.3 (3.3, 22.0) | 0.024 |
| suPAR (ng/mL) | 3.3 (2.3, 4.8) | 3.5 (2.5, 5.2) | 3.2 (2.2, 4.6) | 0.0025 |

For continuous variables median (25th percentile, 75th percentile) is given; hs-TnI: high-sensitivity troponin I; suPAR: soluble urokinase plasminogen activator receptor

**Table S7: Interaction of suPAR attributed risk and sex**

| **Model** | **Sex** | **HR (95% CI)** | **p-value** | **p-interaction** |
| --- | --- | --- | --- | --- |
| Model 1 | Women | 5.57 (3.13, 9.92) | <0.001 | 0.75 |
|  | Men | 6.29 (3.93, 10.06) | <0.001 |  |
| Model 2 | Women | 4.86 (2.55, 9.24) | <0.001 | 0.94 |
|  | Men | 5.01 (3.01, 8.33) | <0.001 |  |
| Model 3 | Women | 4.22 (1.97, 9.04) | <0.001 | 0.86 |
|  | Men | 3.89 (2.28, 6.63) | <0.001 |  |
| Model 4 | Women | 4.06 (1.87, 8.80) | <0.001 | 0.91 |
|  | Men | 3.85 (2.23, 6.66) | <0.001 |  |

Cox regression analyzing the association of logarithmized suPAR levels and overall mortality in women and men. The interaction p-value is given. Model 1: unadjusted; Model 2: adjustment for the variables age and sex; Model 3: adjustment for the variables age, sex, diabetes, smoking, hyperlipoproteinemia and systolic blood pressure; Model 4: adjustment for the variables age, sex, diabetes, smoking, hyperlipoproteinemia, systolic blood pressure and high-sensitivity troponin I. HR: hazard ratio; CI: confidence interval; n.a.: not applicable; suPAR: soluble urokinase plasminogen activator receptor.

**Table S8:** **suPAR levels in different age groups**

|  | **All**  **(N=1314)** | **≤ 60 years (N=568)** | **60-70 years (N=252)** | **≥ 70 years (N=494)** | **p-value** |
| --- | --- | --- | --- | --- | --- |
| Age (years) | 64.0 (51.0, 75.0) | 49.0 (42.0, 55.0) | 66.0 (63.0, 68.0) | 77.0 (73.9, 82.0) | <0.001 |
| Male (%) | 846 (64.4) | 401 (70.6) | 162 (64.3) | 283 (57.3) | <0.001 |
| hs-TnI 0h (ng/L) | 6.8 (3.1, 21.9) | 4.1 (2.0, 11.5) | 8.7 (3.8, 32.5) | 10.6 (5.4, 30.5) | <0.001 |
| suPAR 0h (ng/mL) | 3.3 (2.3, 4.8) | 2.7 (2.0, 3.8) | 3.0 (2.2, 4.5) | 4.3 (3.1, 6.3) | <0.001 |

For continuous variables median (25th percentile, 75th percentile) is given. For binary ones absolute and relative frequencies are shown. hs-TnI: high-sensitivity troponin I; suPAR: soluble urokinase plasminogen activator receptor

**Table S9: Interaction of suPAR attributed risk and different age groups**

| **Model** | **Age groups** | **HR (95% CI)** | **p-value** | **p-interaction** |
| --- | --- | --- | --- | --- |
| Model 1 | ≤ 60 years | 1.58 (0.48, 5.22) | 0.46 | 0.074 |
|  | 60-70 years | 6.58 (2.99, 14.48) | <0.001 |  |
|  | ≥ 70 years | 6.09 (3.67, 10.10) | <0.001 |  |
| Model 2 | ≤ 60 years | 1.41 (0.42, 4.77) | 0.58 | 0.051 |
|  | 60-70 years | 6.97 (3.08, 15.75) | <0.001 |  |
|  | ≥ 70 years | 6.03 (3.52, 10.34) | <0.001 |  |
| Model 3 | ≤ 60 years | 1.14 (0.31, 4.23) | 0.85 | 0.087 |
|  | 60-70 years | 5.72 (2.49, 13.17) | <0.001 |  |
|  | ≥ 70 years | 4.59 (2.52, 8.36) | <0.001 |  |
| Model 4 | ≤ 60 years | 1.20 (0.32, 4.57) | 0.79 | 0.13 |
|  | 60-70 years | 5.68 (2.48, 13.01) | <0.001 |  |
|  | ≥ 70 years | 4.33 (2.34, 8.00) | <0.001 |  |

Cox regression analyzing the association of logarithmized suPAR levels and overall mortality in different age groups. The interaction p-value is given. Model 1: unadjusted; Model 2: adjustment for the variables age and sex; Model 3: adjustment for the variables age, sex, diabetes, smoking, hyperlipoproteinemia and systolic blood pressure; Model 4: adjustment for the variables age, sex, diabetes, smoking, hyperlipoproteinemia, systolic blood pressure and high-sensitivity troponin I. HR: hazard ratio; CI: confidence interval; n.a.: not applicable; suPAR: soluble urokinase plasminogen activator receptor.

**Table S10: Interaction of suPAR attributed risk and different AMI types**

| **Model** | **AMI type** | **HR (95% CI)** | **p-value** | **p-interaction** |
| --- | --- | --- | --- | --- |
| Model 1 | Type 1 | 4.40 (2.63, 7.35) | <0.001 | 0.49 |
|  | Type 2 | 3.07 (1.27, 7.43) | 0.013 |  |
| Model 2 | Type 1 | 4.18 (2.37, 7.38) | <0.001 | 0.37 |
|  | Type 2 | 2.56 (1.02, 6.43) | 0.045 |  |
| Model 3 | Type 1 | 3.79 (2.02, 7.09) | <0.001 | 0.24 |
|  | Type 2 | 1.87 (0.69, 5.12) | 0.22 |  |
| Model 4 | Type 1 | 3.96 (2.04, 7.71) | <0.001 | 0.23 |
|  | Type 2 | 1.84 (0.65, 5.23) | 0.25 |  |

Cox regression analyzing the association of logarithmized suPAR levels and overall mortality in in type 1 and type 2 AMI. The interaction p-value is given. Model 1: unadjusted; Model 2: adjustment for the variables age and sex; Model 3: adjustment for the variables age, sex, diabetes, smoking, hyperlipoproteinemia and systolic blood pressure; Model 4: adjustment for the variables age, sex, diabetes, smoking, hyperlipoproteinemia, systolic blood pressure and high-sensitivity troponin I. HR: hazard ratio; CI: confidence interval; n.a.: not applicable; suPAR: soluble urokinase plasminogen activator receptor.

**Figure S1: Survival analysis according to suPAR quartiles in women and men**

**
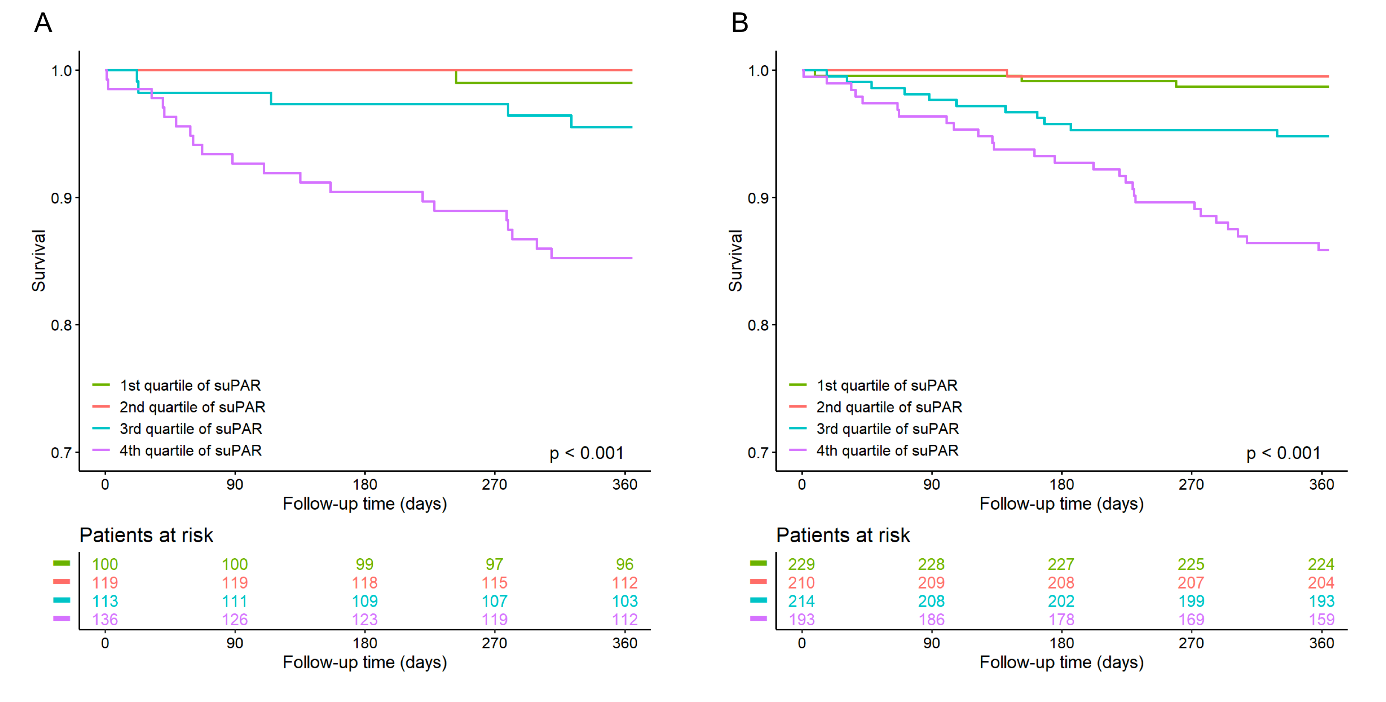
**

Kaplan Meier survival curves for women (A) and men (B) stratified by quartiles of suPAR levels. suPAR: soluble urokinase plasminogen activator receptor
